# Antitubercular drugs induced hepatic oxidative stress and ultrastructural changes in rats

**DOI:** 10.1186/1471-2334-12-S1-P85

**Published:** 2012-05-04

**Authors:** SD Saraswathy, CS Shyamala Devi

**Affiliations:** 1 Department of Biomedical Science, Bharathidasan University, Tiruchirappalli 620 024, India; 2 Department of Biochemistry, University of Madras, Chennai 600 025, India

## Background

As the adverse side effect hepatotoxicity, constitutes an essential part of antituberculosis chemotherapy, the present study was designed to investigate the pathogenesis of antituberculosis (anti-TB) drugs induced hepatic effects in rats with the first-line treatment regimen of isoniazid, rifampicin and pyrazinamide.

## Methods

The rats were divided into three groups (n=6 per group), group I served as a control, group II received orally combination of isoniazid (15mg/kg body weight), rifampicin (20mg/kg body weight) and pyrazinamide (35mg/kg body weight) daily for 45 days and group III received simultaneously *Silymarin* (50mg/kg body weight) and combination of anti-TB drugs at the above mentioned dosages for 45 days. After the experimental period, the levels of malondialdehyde (MDA, oxidative stress marker) and lipid profile was evaluated in serum. The data were analyzed by Duncan’s multiple range tests. The pathological and morphological changes were examined histologically and electron microscopically.

## Results

The rats administered anti-TB drugs alone, showed a significantly increase in serum MDA levels and lipid profile (p<0.001). Histopathological features of group II rats showed inflammatory cell infiltration and spotty necrosis. The electron micrograph results indicated kupffer cell hyperplasia, swollen mitochondria and loss of cell architecture. Co-administration of *Silymarin* significantly decreased anti-TB drugs-induced changes in serum MDA levels, lipids (p<0.001) and retained the liver integrity.

## Conclusions

The anti-TB drugs can induce hepatic oxidative stress and the level of serum MDA may be a more sensitive biomarker for monitoring drug-induced hepatotoxicity. Hepatoprotective compounds with antioxidant potential can be supplemented to prevent anti-TB drugs induced cellular oxidative stress.

